# Supplementary material for: Motoric Cognitive Risk Syndrome, Subtypes and 8-Year All-Cause Mortality in Aging Phenotypes: The Salus in Apulia Study
Source: Brain Sci. 2022 Jun 29;12(7):861. doi: 10.3390/brainsci12070861 (PMC9313038; doi:10.3390/brainsci12070861)
Supplement: Supplementary file 1 [file brainsci-12-00861-s001.zip › brainsci-1690230-supplementary.pdf]

Variables	Overall Sample	No MCR	MCR	p	Effect Size*	No MCR	MCR Global Function	p	Effect Size*	No MCR	MCR Structured SCC	p	Effect Size*	NO MCR	MCR SCC & Global Function	p	Effect Size*
Proportions (%)		1026 (90.20)	112 (9.80)			1097 (96.40)	41 (3.60)			1099 (96.60)	39 (3.40)			1117 (98.20)	21 (1.80)		
Age (years)	74.51 ± 6.11	74.4 ± 6.09	75.57 ± 6.16	0.06	-0.19 (-0.39, 0.01)	74.39 ± 6.08	77.83 ± 5.97	<0.01	**-0.57 (-0.88, -0.25)**	74.39 ± 6.07	77.85 ± 6.12	<0.01	**-0.57 (-0.89, -0.25)**	74.47 ± 6.12	76.57 ± 4.71	0.06	-0.34 (-0.78, 0.09)
Sex																	
Male	552 (48.50)	504 (49.10)	48 (42.90)	0.24	6.27 (-3.40, 15.93)	536 (48.90)	16 (39.00)	0.21	9.84 (-5.39, 25.06)	535 (48.70)	17 (43.60)	0.53	5.09 (-10.75, 20.93)	543 (48.60)	9 (42.90)	0.60	5.76 (-15.61, 27.12)
Female	586 (51.50)	522 (50.90)	64 (57.10)			561 (51.10)	25 (61.00)			564 (51.30)	22 (56.40)			574 (51.40)	12 (57.10)		
BMI (Kg/m^2)	28.37 ± 4.78	28.31 ± 4.73	28.95 ± 5.22	0.25	-0.13 (-0.33, 0.06)	28.3 ± 4.73	30.43 ± 5.66	0.01	-0.45 (-0.76, -0.14)	28.32 ± 4.74	29.86 ± 5.56	0.06	-0.32 (-0.64, 0.01)	28.36 ± 4.75	29.15 ± 6.32	0.83	-0.17 (-0.60, 0.27)
Education (years)	7.11 ± 3.76	7.2 ± 3.72	6.29 ± 4.05	<0.01	0.24 (0.05, 0.44)	7.23 ± 3.75	4.02 ± 2.44	<0.01	**1.31 (0.99, 1.63) §**	7.11 ± 3.73	6.97 ± 4.69	0.15	0.04 (-0.28, 0.36)	7.17 ± 3.75	3.67 ± 2.31	<0.01	**0.94 (0.51, 1.37)**
Smokers (Yes)	91 (8.00)	84 (8.20)	7 (6.20)	0.47	-1.94 (-6.72, 2.85)	90 (8.20)	1 (2.4)	0.24	**-5.77 (-10.76, -0.77)**	90 (8.20)	1 (2.60)	0.36	**-5.63 (-10.84, -0.41)**	91 (8.10)	--	0.40	
MMSE	26.94 ± 2.75	27 ± 2.72	26.42 ± 2.98	0.04	0.21 (0.02, 0.41)	27.13 ± 2.59	21.81 ± 1.51	<0.01	**3.54 (3.19, 3.88) §**	26.96 ± 2.74	26.49 ± 2.86	0.34	0.17 (-0.15, 0.49)	27.05 ± 2.66	21.49 ± 1.56	<0.01	**3.57 (3.11, 4.02) §**
RAVLTi	35.03 ± 7.48	35.0 ± 7.35	35.0 ± 8.60	0.52	0.01 (-0.18, 0.21) §	35.26 ± 7.43	29.05 ± 6.46	<0.01	**0.84 (0.53, 1.15)**	35.1 ± 7.47	33.21 ± 7.83	0.16	0.25 (-0.07, 0.57)	35.19 ± 7.42	26.89 ± 6.24	<0.01	**1.12 (0.69, 1.55)**
RAVLTd	6.83 ± 2.57	6.85 ± 2.58	6.68 ± 2.55	0.72	0.07 (-0.13, 0.26)	6.89 ± 2.54	5.44 ± 3.07	<0.01	0.56 (0.25, 0.88)	6.85 ± 2.57	6.3 ± 2.62	0.29	0.22 (-0.10, 0.54)	6.87 ± 2.56	4.84 ± 2.52	<0.01	**0.79 (0.36, 1.23)**
CDT	10.63 ± 2.76	10.7 ± 2.74	9.96 ± 2.87	<0.01	0.27 (0.08, 0.47)	10.73 ± 2.69	8.02 ± 3.35	<0.01	**0.81 (0.49, 1.12) §**	10.66 ± 2.74	9.61 ± 3.01	0.02	0.38 (0.06, 0.70)	10.69 ± 2.72	7.43 ± 3.12	0.02	**1.20 (0.76, 1.63)**
Slowness (Yes)	232 (20.40)	120 (11.70)	112 (100.00)	<0.01	**88.30 (86.34, 90.27)**	191 (17.40)	41 (100.00)	<0.01	**82.59 (80.34, 84.83)**	193 (17.60)	39 (100.00)	<0.01	**82.44 (80.19, 84.69)**	91 (8.10)	21 (100.00)	<0.01	**81.11 (78.81, 83.41)**
Interleukin 6 (pg/ml)	3.28 ± 5.1	3.14 ± 4.92	4.58 ± 6.36	<0.01	-0.23 (-0.42, -0.03) §	3.13 ± 4.76	7.29 ± 9.99	<0.01	-0.42 (-0.73, -0.10) §	3.24 ± 5	4.47 ± 7.26	0.19	-0.24 (-0.56, 0.08)	3.2 ± 4.9	7.95 ± 10.75	0.29	-0.44 (-0.87, -0.01) §
Tumor Necrosis Factor alpha (pg/ml)	2.80 ± 3.85	2.77 ± 3.76	3.08 ± 4.58	0.10	-0.08 (-0.28, 0.11)	2.78 ± 3.86	3.42 ± 3.46	<0.01	-0.17 (-0.48, 0.15)	2.81 ± 3.9	2.64 ± 1.85	0.26	0.04 (-0.28, 0.36)	2.8 ± 3.88	2.82 ± 1.07	0.06	-0.06 (-0.44, 0.43)
C Reactive Protein (mg/dl)	0.58 ± 0.81	0.57 ± 0.83	0.64 ± 0.71	0.06	-0.08 (-0.28, 0.11)	0.58 ± 0.82	0.54 ± 0.59	0.50	0.05 (-0.26, 0.36)	0.57 ± 0.82	0.75 ± 0.69	0.06	-0.21 (-0.53, 0.11)	0.58 ± 0.82	0.62 ± 0.73	0.66	-0.05 (-0.48, 0.38)
FBG (Fasting Blood Glucose) (mg/dl)	105.19 ± 28.79	104.7 ± 27.17	109.69 ± 40.6	0.17	-0.17 (-0.37, 0.02)	105.16 ± 28.98	106.07 ± 23.41	0.80	-0.03 (-0.34, 0.28)	104.75 ± 27.16	117.49 ± 57.67	0.08	-0.04 (-0.35, 0.27) §	105.19 ± 28.92	105.1 ± 20.73	0.82	0.03 (-0.43, 0.44)
Total Cholesterol (mg/dl)	184.83 ± 36.84	184.86 ± 36.38	184.54 ± 40.92	0.93	0.01 (-0.19, 0.20)	185.18 ± 36.75	175.49 ± 38.39	0.10	0.26 (-0.05, 0.58)	184.81 ± 36.76	185.49 ± 39.35	0.98	-0.02 (-0.34, 0.30)	184.98 ± 36.79	176.86 ± 39.19	0.41	0.22 (-0.21, 0.65)
HDL Cholesterol (mg/dl)	49.42 ± 13	49.30 ± 12.80	50.10 ± 15.00	0.90	-0.05 (-0.24, 0.15)	49.52 ± 12.95	46.76 ± 14.02	0.11	0.21 (-0.10, 0.52)	49.43 ± 12.87	49.1 ± 16.42	0.44	0.20 (-0.11, 0.51)	49.44 ± 13	48.38 ± 13.14	0.60	0.08 (-0.35, 0.51)
LDL Cholesterol (mg/dl)	114.03 ± 31.2	114.00 ± 31.00	111.00 ± 33.40	0.40	0.10 (-0.09, 0.30)	114.27 ± 31.14	107.61 ± 32.67	0.15	0.21 (-0.10, 0.53)	114.11 ± 31.2	111.54 ± 31.68	0.85	0.08 (-0.24, 0.40)	114.12 ± 31.12	109.19 ± 36	0.52	0.16 (-0.27, 0.59)
Triglycerides (mg/dl)	103.04 ± 60.19	103.1 ± 59.98	102.53 ± 62.36	0.48	0.01 (-0.19, 0.20)	102.96 ± 60.5	105.37 ± 51.77	0.45	-0.04 (-0.35, 0.27)	102.58 ± 59.76	116.18 ± 70.99	0.40	-0.23 (-0.55, 0.09)	103.24 ± 60.57	92.57 ± 33.26	0.86	0.18 (-0.25, 0.61)
SBP (mmHg)	133.45 ± 14.55	133.19 ± 14.59	135.85 ± 14.03	0.06	0.02 (-0.17, 0.22)	133.31 ± 14.56	137.2 ± 14.14	0.06	-0.27 (-0.58, 0.05)	133.39 ± 14.58	135.13 ± 13.93	0.45	-0.12 (-0.44, 0.20)	133.39 ± 14.57	136.9 ± 13.46	0.19	-0.24 (-0.67, 0.19)
DBP (mmHg)	78.02 ± 7.92	78.04 ± 8.01	77.86 ± 7.09	0.75	-0.18 (-0.38, 0.01)	78 ± 7.92	78.66 ± 8.06	0.65	-0.27 (-0.58, 0.05)	78.07 ± 7.96	76.67 ± 6.62	0.19	0.18 (-0.14, 0.50)	78.01 ± 7.93	78.81 ± 7.4	0.54	-0.10 (-0.53, 0.33)
MACQ (score)	22.29 ± 1.99	22.2 ± 1.97	22.8 ± 2.15	<0.01	-0.25 (-0.45, -0.06) §	22.28 ± 1.99	22.41 ± 2.05	0.71	-0.07 (-0.38, 0.24)	22.15 ± 1.88	26.08 ± 1.35	<0.01	**-2.11 (-2.44, -1.78)**	22.27 ± 1.98	23.19 ± 2.4	0.04	-0.46 (-0.90, -0.03)
SCC (Yes)	545 (47.90)	433 (42.20)	112 (100.00)	<0.01	**57.80 (54.78, 60.82) §**	524 (47.80)	21 (52.10)	0.60	3.45 (-12.13, 19.04)	528 (47.10)	27 (69.20)	<0.01	**22.10 (7.31, 36.88)**	524 (46.90)	21 (100.00)	<0.01	**53.09 (50.16, 56.02)**
TMTA (s)	75.70 ± 42.84	73.10± 40.60	99.20 ± 54.20	<0.01	-0.48 (-0.68, -0.28) §	73.36 ± 39.78	138.29 ± 68.17	<0.01	**-1.58 (-1.90, -1.26) §**	74.67 ± 42.18	104.59 ± 51.33	<0.01	**-0.95 (-1.27, -0.64) §**	74.37 ± 41.52	146.48 ± 52.75	<0.01	**-1.37 (-1.80, -0.93) §**
TMTB (s)	163.91 ± 99.22	160 ± 97.10	200 ± 111.00	<0.01	-0.36 (-0.55, -0.16) §	161 ± 97.84	241.85 ± 105.36	<0.01	**-0.82 (-1.14, -0.51)**	162.78 ± 98.79	195.79 ± 107.18	0.05	-0.33 (-0.65, -0.01)	162.09 ± 98.06	260.48 ± 114.63	<0.01	**-1.00 (-1.43, -0.57)**
Time of observation (months)	62.73 ± 21.37	62.64 ± 21.36	63.56 ± 21.53	0.39	-0.04 (-0.24, 0.15)	63.07 ± 21.26	53.66 ± 22.53	0.01	**0.44 (0.13, 0.75)**	62.74 ± 21.35	62.38 ± 22.15	0.82	0.02 (-0.30, 0.34)	62.86 ± 21.32	55.76 ± 23	0.23	0.33 (-0.10, 0.76)
Mean Survival Time (months)	85.50 ± 19.57	85.50 ± 19.57	83.40 ± 20.94	0.10		85.60 ± 19.27	77.20 ± 28.42	<0.01		85.50 ± 19.49	80.20 ± 3.87	0.06		85.50 ± 19.49	80.20 ± 24.16	0.20	

**Cohen' delta effect size where not otherwise specified, § Glass' delta effect size and prevalence differences for categorical variables*
All data are shown as mean ± sd for continuous variables and as n (%) for proportions .
Medium to large effect size (>0.5) were highlighted in bold

MMSE: Mini Mental Statement Examination; RAVLT: Rey Auditory Verbal Learning Test (i, immediate; d, delayed); CDT: Clock Drawing Test; MAC-Q: Memory Assessment Clinic-Q; SCC: Subjective Cognitive Complaint; TMT: Trail Making Test
